# Soil nitrate reducing processes – drivers, mechanisms for spatial variation, and significance for nitrous oxide production

**DOI:** 10.3389/fmicb.2012.00407

**Published:** 2012-12-18

**Authors:** Madeline Giles, Nicholas Morley, Elizabeth M. Baggs, Tim J. Daniell

**Affiliations:** ^1^Institute of Biological and Environmental Sciences, School of Biological Sciences, University of AberdeenAberdeen, UK; ^2^Ecological Sciences, The James Hutton InstituteDundee, UK

**Keywords:** denitrification, dissimilatory nitrate reduction to ammonium, nitrous oxide, functional diversity, spatial heterogeneity, linkage between community structure and activity

## Abstract

The microbial processes of denitrification and dissimilatory nitrate reduction to ammonium (DNRA) are two important nitrate reducing mechanisms in soil, which are responsible for the loss of nitrate (${\text{NO}}_{3}^{-}$) and production of the potent greenhouse gas, nitrous oxide (N_2_O). A number of factors are known to control these processes, including O_2_ concentrations and moisture content, N, C, pH, and the size and community structure of nitrate reducing organisms responsible for the processes. There is an increasing understanding associated with many of these controls on flux through the nitrogen cycle in soil systems. However, there remains uncertainty about how the nitrate reducing communities are linked to environmental variables and the flux of products from these processes. The high spatial variability of environmental controls and microbial communities across small sub centimeter areas of soil may prove to be critical in determining why an understanding of the links between biotic and abiotic controls has proved elusive. This spatial effect is often overlooked as a driver of nitrate reducing processes. An increased knowledge of the effects of spatial heterogeneity in soil on nitrate reduction processes will be fundamental in understanding the drivers, location, and potential for N_2_O production from soils.

## INTRODUCTION

**FIGURE 1:**
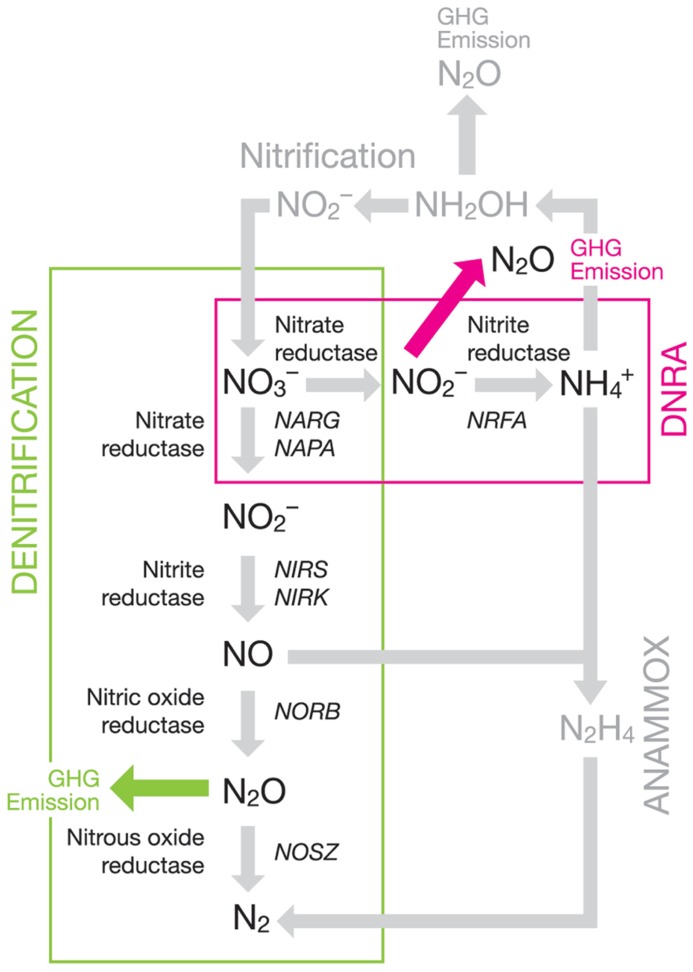
**Partial soil nitrogen cycling pathways with an emphasis on denitrification and DNRA showing the enzymes involved and genes commonly used as markers**.

Anthropogenic effects on the global nitrogen cycle have disrupted the biogeochemical processes involved (Galloway and Cowling, 2002; Gruber and Galloway, 2008; Rockstrom et al., 2009). This is due, in part, to an 800% increase in nitrogen fertilizer use facilitated by the discovery of the Haber–Bosch process, and the poor efficiency with which these fertilizers are used by crop plants (Canfield et al., 2010). The downstream effects of this low utilization have led to environmental issues such as eutrophication of water bodies through nutrient leaching and increased production of nitrous oxide (N_2_O), an important greenhouse gas. N_2_O has a global warming potential around 300 times greater than that of carbon dioxide (CO_2_) over a 100-year period (Forster et al., 2007), as well has having the potential to damage the ozone layer (Cicerone, 1987). Agricultural soils are believed to contribute as much as 60% of global N_2_O emissions (Smith et al., 2007), primarily through microbially driven soil processes such as denitrification (Stehfest and Bouwman, 2006) and dissimilatory nitrate reduction to ammonium (DNRA). Denitrification and DNRA are two of several important processes that are responsible for nitrogen cycling in soil. Other key nitrogen transforming processes in soil include nitrification (Stark and Hart, 1997; De Boer and Kowalchuk, 2001; Brown et al., 2012), which is the oxidative conversion of ammonium to nitrate, and anaerobic ammonium oxidation (Anammox) which oxidizes ammonium to nitrogen gas (N_2_) gas using nitrite (${\text{NO}}_{2}^{-}$) as the electron acceptor (Humbert et al., 2010; Ishii et al., 2011; Hu et al., 2011; Zhu et al., 2011). Nitrification, denitrification, and DNRA are all capable of producing N_2_O (**Figure 1**). Both denitrification and DNRA are processes that reduce nitrate (${\text{NO}}_{3}^{-}$) through various intermediate steps to N_2_ and ammonium (${\text{NH}}_{4}^{+}$), respectively, and are the only soil microbial processes capable of both removing soil ${\text{NO}}_{3}^{-}$ and producing N_2_O. Denitrification produces N_2_O when abiotic conditions or gene complement prevent its reduction to N_2_ and DNRA releases N_2_O as a by-product of the reduction process.

As denitrification and DNRA are microbial processes both the soil nitrate reducing community and the abiotic conditions in soil are important in controlling the production of N_2_O. The strong gradients in abiotic factors over short distances make it critical to understand the effects of the complex soil environment on both the soil ${\text{NO}}_{3}^{-}$ reducing communities and abiotic conditions in soil. This complex soil environment will affect the spatial and temporal location of both denitrification and DNRA (Enwall et al., 2010; Keil et al., 2011). It is important to understand that a complex and interactive number of factors including those that regulate relevant gene expression and drive the wider ecology of the microorganisms are involved. In particular, it is critical to understand how these factors vary both spatially and temporally in soil. A greater understanding of the dynamics of denitrification and DNRA may allow the development of more effective mitigation strategies focused on areas representing important sources of N_2_O.

This review aims to summarize current knowledge of both the abiotic factors and microbial communities involved in nitrate reducing processes in soil systems. It aims to assess how this affects our understanding of the location and activity of nitrate reduction in soil, and the importance of spatial heterogeneity. It highlights the attempts to understand linkage between environmental controls, process flux, and the communities responsible, and further explores the possible reasons behind the observed lack of explicit links between these factors in soil. This review highlights key gaps in our knowledge that currently preclude an understanding of any existing linkages and emphasizes denitrification because of the large body of work published on this process.

## DENITRIFICATION

Denitrification is a facultative anaerobic reaction that sequentially reduces ${\text{NO}}_{3}^{-}$ to N_2_ (**Figure 1**) via ${\text{NO}}_{2}^{-}$, and the gases nitric oxide (NO) and N_2_O under oxygen (O_2_) limiting conditions (Robertson and Tiedje, 1987;Bremner, 1997). It allows the maintenance of respiration (Zumft, 1997) when O_2_ is limiting through the use of the N-oxides as terminal electron acceptors, although some steps may also occur under oxic conditions (Richardson, 2000; Morley et al., 2008). The process is catalyzed by a number of enzymes including nitrate reductase, nitrite reductase, nitric oxide reductase, and nitrous oxide reductase, which are encoded by the genes *narG*, *nirS/nirK*, *norB*, and *nosZ*, respectively and are most commonly used markers to understand the denitrifying community although other nitrate reducing genes also occur. The different enzymes are to an extent modular allowing intermediate products to accumulate during denitrification. The exception to this is nitrite and nitric oxide reductases, which are expressed co-ordinately ensuring that ${\text{NO}}_{2}^{-}$ and NO do not accumulate as both are cytotoxic. Accumulation of intermediates can arise due to either differential enzymatic rates (Betlach and Tiedje, 1981; Thomsen et al., 1994), abiotic factors inhibiting one or more enzymes (Burford and Bremner, 1975; Bateman and Baggs, 2005; Van den Heuvel et al., 2011), differential transcription of functional genes (Bergaust et al., 2008; Bakken et al., 2012), or can be genomic (lack of a functional gene within genome; Philippot et al., 2011). Truncation of the denitrification process is a major factor influencing soil N_2_O emissions. In addition, the microbial community present likely affects both the rate of production and the gaseous products yielded by denitrification, primarily controlled through the presence/absence, abundance and activation of the genes responsible. A number of environmental factors are known to control the rate of denitrification including, O_2_ and water content of soils (Bateman and Baggs, 2005), ${\text{NO}}_{3}^{-}$ (Smith and Tiedje, 1979; Klemedtsson et al., 1991), carbon (Burford and Bremner, 1975), pH (Simek and Cooper, 2002; Van den Heuvel et al., 2011), and temperature (Wolf and Brumme, 2002).

### OXYGEN

As denitrification functions under anoxic conditions, soil O_2_ availability is an important driver. Aside from O_2_ partial pressures in the gas phase, water is the most important regulatory factor of soil aeration as it presents a barrier to rapid O_2_ diffusion resulting in a strong link between O_2_ availability and soil water content (Smith, 1990). Soil texture and biological activity also play a crucial role in O_2_ availability, creating O_2_ gradients as a result of diffusion and aerobic respiration (Tiedje, 1988).

O_2_ reduces the activity of denitrification enzymes by regulating the flow of electrons, suppressing the expression of structural denitrifying genes (Berks et al., 1995), and inhibition of ${\text{NO}}_{3}^{-}$ uptake systems (Hernandez and Rowe, 1987). N_2_O reductase is the most sensitive to O_2_ inhibition (Knowles, 1982; Morley et al., 2008). Consequently, N_2_O may still be the dominant product of denitrification in soils a few days after rainfall or irrigation as O_2_ availability is still decreasing due to existing pools of active nitrite reductase (Clayton et al., 1997; Dobbie and Smith, 2001; Webb et al., 2004). The importance of water to denitrification was demonstrated by Weier et al. (1993) who found that increasing water filled pore space (WFPS) increased denitrification rates but also induced greater N_2_ production.

### NITROGEN

 The presence of a suitable form of nitrogen is vital for the occurrence of denitrification as it is used as a terminal electron acceptor when O_2_ is limiting. The application of a variety of N fertilizers to soils has been shown to stimulate denitrification (Clayton et al., 1997; Webb et al., 2004) and lead to the production of both N_2_ and N_2_O. Where ${\text{NO}}_{3}^{-}$ is limiting, for example in the rhizosphere where there is competition between plants and microorganisms, reduced N_2_O emissions have been observed (Duxbury et al., 1982). High ${\text{NO}}_{3}^{-}$ concentrations can lower the reduction of N_2_O to N_2_ (Firestone et al., 1979; Gaskell et al., 1981; Weier et al., 1993). Blackmer and Bremner (1978) suggested that N_2_O dominated emission occurred when N–${\text{NO}}_{3}^{-}$ concentrations were over 10 μg g^-^^1^ soil, as at these concentrations ${\text{NO}}_{3}^{-}$ is preferentially reduced over N_2_O. This may be explained by the relative low energy yield gained during N_2_O reduction in comparison to other nitrogen oxides (Koike and Hattori, 1975).

Other forms of organic and inorganic N may also play a role in denitrification as co-substrates in co-denitrification. This occurs where one N atom from NO or N_2_O combines with an atom from another N source (a co-substrate) forming a hybrid product (Su et al., 2004). The range of N-containing compounds that can be used as co-substrates is potentially large but there still remains uncertainty about the range of compounds that can act as potential co-substrates. Laughlin and Stevens (2002) were able to demonstrate co-denitrification in grassland soils, however, it is still unclear the importance of this process in other ecosystems.

### CARBON

As denitrification maintains respiration under low O_2_ conditions the availability of C is critical for activity and it is commonly limited in soil either through location or chemical form. Carbon substrate degradation pathways and the TCA cycle produce the reducing equivalent NADH, providing a source of electrons for denitrifying enzymes (Richardson, 2000). Many studies have shown that C can affect the ability of soils to denitrify (Burford and Bremner, 1975; Dendooven et al., 1996; Mounier et al., 2004; Dodla et al., 2008; Henry et al., 2008). Observed responses are variable because of differences in soil conditions, C compounds, and the quantity of C added. The presence of readily decomposable organic C substrates have been shown to decrease N_2_O:N_2_ ratios compared to C-limited soils thus reducing greenhouse gas emission (Firestone and Davidson, 1989; Weier et al., 1993). As well as changing denitrification product ratios, the presence of labile organic C substrates in soils has been shown to stimulate denitrification rates (Azam et al., 2002), with the effects dependent upon both the quantity and form of C. The quantity of C has effects that can be either direct, through influence on the denitrifying organisms, by supplying a source of reductant, or indirect, by the stimulation of soil heterotrophic respiration lowering O_2_ partial pressure in soils and thus creating anaerobic conditions favorable for denitrification.

The form of C also affects denitrification, for example, Henry et al. (2008) applied artificial root exudates comprised of different combinations of sugars, organic acids, and amino acids, to soil microcosms. This showed that a high proportion of sugars produced lower N_2_O:N_2_ ratios due to N limitation. The C compound may also affect both the rate of denitrification (Lorrain et al., 2004) and the amount of C required to denitrify a specific amount of ${\text{NO}}_{3}^{-}$ (Christensson et al., 1994). These differences may arise because C substrates regulate the same enzyme differently dependent on concentration, or the same substrate may affect different reductases differently. A study by Morley and Baggs (2010) demonstrated a significant difference in the effects of C compound on the quantity of N_2_O and N_2_ produced from an agricultural soil, although the effects varied with O_2_ headspace concentration. Other studies have found similar results, however, whether the effects of C are predominately biochemical or act indirectly through alteration of bacterial community composition remains unclear (Jacobson and Alexander, 1980; Dendooven et al., 1996; Murray et al., 2004; Miller et al., 2008). Relatively labile C compounds, including those used in the above studies, are readily utilized by soil microorganisms and have a half-life of minutes to hours (Paterson et al., 2008). Interactions between factors are also important, Morley and Baggs (2010), for example, demonstrated significant interactions between O_2_ concentration and C compound in the regulation of denitrification enzymes, resulting in contrasting N_2_O:N_2_ product ratios.

Members of the soil microbial community other than denitrifiers can be affected by the addition of C compounds, which may affect denitrification. Shi et al. (2011) showed an increase in the overall taxon richness of soil samples amended with between 0.1 and 0.3 mg C g^–^^1^ dry weight soil a day for 15 days, in the form of a mixture of sugar and organic acids. The effect of glucose addition on denitrifying genes has been investigated by measuring the abundance of *nosZ* after C addition. The results of such studies have been variable with both increases in *nosZ* (Henderson et al., 2010) and no change in *nosZ* abundance (Miller et al., 2008) observed. Differences in experimental time scales and the soils studied may help to account for such contrasting results but these examples demonstrate how the response of denitrification is highly context specific. It is therefore important not to rely on single or a low numbers of studies to provide conclusive understanding to variables such as C. Other more complex mixtures of compounds have also been tested. For example, Mounier et al. (2004) investigated the effect of maize mucilage, a complex rhizodeposit containing many forms of C, on denitrifiers. Here the dominant *narG* RFLP families did not vary between treatments with and without amendments but the abundance of *narG* did increase.

### pH

pH has been demonstrated to be an important control on denitrification through enzyme sensitivity (Firestone et al., 1980; Van den Heuvel et al., 2011). A pH of between 7.0 and 8.0 has been suggested as optimum for denitrification (Knowles, 1982). Soil pH is a major driver of denitrifier N_2_O:N_2_ ratios and numerous studies have shown that the dominant product of denitrification is N_2_O under acidic conditions due to the severe impairment of N_2_O reductase (Simek and Cooper, 2002; Liu et al., 2010; Van den Heuvel et al., 2011). Interestingly, Bergaust et al. (2010) showed no effect of pH on the transcription of the N_2_O reductase functional gene (*nosZ*) in *Paracoccus denitrificans*. Only the enzymatic rate was reduced at low pH suggesting that environmental pH has a direct effect post-translational on the assembly or activity of a functional N_2_O reductase. pH has also been shown to affect both the community structure and the proportional contributions of different microbial groups to N_2_O production in arable soils (Enwall et al., 2005; Baggs et al., 2010; Herold et al., 2012). Additionally, indirect effects of pH include reduction of available mineral nitrogen and organic carbon at low pH (Simek and Cooper, 2002; Baggs et al., 2010) and the role of pH in shaping microbial community structure in soils across continental scales from numerous ecosystems (Fierer and Jackson, 2006).

## DISSIMILATORY NITRATE REDUCTION TO AMMONIUM

A second, lesser characterized, nitrate reducing process is that of DNRA, or nitrate ammonification, in which ${\text{NO}}_{3}^{-}$ is reduced to ${\text{NO}}_{2}^{-}$ and ${\text{NH}}_{4}^{+}$, with N_2_O produced at the ${\text{NO}}_{2}^{-}$reduction stage (**Figure 1**) as a by-product (Kelso et al., 1997; Rutting et al., 2011; Streminska et al., 2012). The enzyme for the reduction of ${\text{NO}}_{2}^{-}$is coded by *nrfA* gene. The process itself is a respiratory mechanism that derives energy from the generation of a proton-motive force across the membrane (Kraft et al., 2011). N_2_O produced by DNRA cannot be further reduced during this process, so it would be environmentally advantageous for DNRA and denitrification to be closely coupled. It is possible that in some cases DNRA N_2_O production may have been wrongly attributed to denitrification, though DNRA’s contribution may be small. A study by Inselsbacher et al. (2010) found that DNRA did not make a significant contribution to the ammonium pools in two agricultural soils under the conditions studied. Similarly, Silver et al. (2005) found that DNRA only represented 3% of N mineralization in a tropical forest system. Despite the relatively limited levels of DNRA it still provides a means of preventing the loss of N from systems by converting ${\text{NO}}_{3}^{-}$ to ${\text{NH}}_{4}^{+}$. Both bacteria and fungi have been found to be capable of carrying out DNRA (Takaya, 2002; Philippot, 2005) and the *nrfA* gene has been shown to be present in a wide variety of bacteria (Smith et al., 2007).

Dissimilatory nitrate reduction to ammonium is less sensitive to O_2_ fluctuations than denitrification (Fazzolari et al., 1998), but generally occurs under anaerobic conditions. While information on the effects of C or nitrogen on DNRA independently of each other is limited, the C:${\text{NO}}_{3}^{-}$ ratio is considered an important control on the process. Initially it was suggested that DNRA was more likely to be important under conditions of limited C availability (Cole and Brown, 1980), however it has been suggested that in culture (Smith, 1982) and in soil (Schmidt et al., 2011) the greatest reduction of ${\text{NO}}_{3}^{-}$ occurred at high C:N ratios. Tyson et al. (1994) found using *Escherichia coli* in culture, that *nrfA* expression could be repressed in the presence of ${\text{NO}}_{3}^{-}$, resulting in accumulation of ${\text{NO}}_{2}^{-}$. Denitrification as a reduction pathway is therefore believed to be favored where ${\text{NO}}_{3}^{-}$ concentrations are high (Stevens and Laughlin, 1998), pH has also found to be a control on DNRA with Stevens and Laughlin (1998) showing that greater reduction of ${\text{NO}}_{3}^{-}$ by DNRA occurred at higher pHs in pasture soils.

## ORGANISMS INVOLVED IN DENITRIFICATION

A wide range of organisms including archaea, bacteria, and fungi and are known to be capable of denitrification. In soil both fungi and bacteria are known to denitrify (Laughlin and Stevens, 2002; Dandie et al., 2008; Herold et al., 2012). Archaeal denitrification activity has been demonstrated in culture, but little is known about their ability to denitrify in natural systems (Hayatsu et al., 2008). Bacteria remain the best understood of the denitrifiers, with many studies investigating denitrification both in culture and natural systems. Denitrifying bacteria are geographically widespread (Gamble et al., 1977), and the ability to denitrify has been found in phylogenetically diverse organisms with a range of environmental tolerances (Zumft, 1997; Jones et al., 2008). Not all denitrifying bacteria contain all the genes necessary for N_2_ to be produced. An estimated one third of genomes containing *nirK/nirS* and *norB* do not contain the *nosZ *gene required to produce N_2_ (Jones et al., 2008). Henry et al. (2006) quantified both small subunit *16srRNA* and *nosZ* in a range of soils, using real-time PCR (RT-PCR) and found, that only 5–6% of microorganisms quantified using *16srRNA* were likely to contain *nosZ*.

Additionally, many fungi are capable of denitrification (Takaya, 2009), and have a significant role in nitrogen cycling (Laughlin and Stevens, 2002; Seo and DeLaune, 2010; Herold et al., 2012). Mycorrhizal fungi have provided an opportunity to assess the effects of fungi in soil through exclusion systems and comparisons between colonized and non colonized plants. Amora-Lazcano et al. (1998) compared the abundance of denitrifiers, ammonium oxidizers, and ammonifying bacteria between maize plants with and without arbuscular mycorrhizal fungi. The study found that after 30 days the presence of arbuscular mycorrhizal fungi reduced the population size of both bacterial denitrifiers and ammonifying organisms in soil, which may have been driven by the alteration in soil conditions created by the presence of arbuscular mycorrhizal fungi. Indirect evidence also exists that ectomycorrhiza fungi can affect N_2_O emissions. The exclusion of ectomycorrhiza fungi from soil was shown by Ernfors et al. (2011) to increase N_2_O emissions; a result they attributed to either increased water or N content of the soil when ectomycorrhiza fungi were excluded. Direct effects have also been demonstrated for some ectomycorrhizal taxa, with studies demonstrating that some ectomycorrhiza fungi are capable of growth on ${\text{NO}}_{3}^{-}$ and maintain the genes necessary for nitrate reduction (Nygren et al., 2008). Further to this the ectomycorrhiza fungi *Paxillus involutus *and *Tylospora fibrillosa* were shown by Prendergast-Miller et al. (2011) to be capable of N_2_O production.

### LINKS BETWEEN ENVIRONMENTAL CONDITIONS, DENITRIFIER COMMUNITIES, AND DENITRIFICATION RATES

Field studies have successfully linked a range of environmental variables to the production of N_2_O by denitrification. Factors such as soil moisture content, C availability, and soil ${\text{NH}}_{4}^{+}$ content have all been found to be useful predictors of denitrification (Davis et al., 2008; Gillam et al., 2008; Vilain et al., 2012). Despite this, microcosm experiments have shown variation in responses to the same environmental variables, such as C addition, with the quantity of N_2_O production varying between studies by as much as 997 mg N–N_2_O kg^-^^1^ of dry weight soil (**Table 1**). Even when the addition of the same C compounds are compared, e.g., glucose, there are large differences in the production of N_2_O and N_2_. For example, a study by Morley and Baggs (2010) found only N_2_ to be produced with glucose addition, while Murray et al. (2004) found both N_2_O and N_2_. Comparisons between studies must be made cautiously because of varying soil types and experimental conditions. This highlights our lack of complete understanding about the controls and drivers of denitrification. Moisture content, temperature, and pH were similar between many of the microcosm studies on denitrification, but C:N ratio and soils used varied greatly. These differences in soil conditions will also lead to contrasting microbial community structures and reinforces the need to understand the link between environmental variables, soil processes, and the microbial community responsible for denitrification.

**Table 1:** Studies measuring the effects of low molecular weight carbon compounds on the production of N_2_O from soils.

Paper ecosystem	Compound added	Amount C added (mg kg^−1^ dry weight soil)	Amount N added (mg kg^−1^ dry weight soil)	C:N	N_2_O–N (mg kg^−1^ dry weight soil)	N_2_–N (mg kg^−1^ dry weight soil)
Henderson etal. (2010)	Glucose	1000.00	500.00	2.00	94.00	n/a
Arable						
Murray etal. (2004)	Glucose	110.00	66.00	1.67	1000.00	36.70
Grassland	Cellulose	110.00	66.00	1.67	466.70	0.00
	Starch	110.00	66.00	1.67	683.30	0.00
Senbayram etal. (2012)	Sucrose	500.00	110.00	4.55	26.26	n/a
Arable						
Miller etal. (2008)	Glucose	250.00	50.00	5.00	3.00	n/a
Arable						
Morley and Baggs (2010)	Glucose	960.00	66.00	14.54	0.00	9.60
Grassland	Mannitol	960.00	66.00	14.54	0.00	12.80
	Glutamic acid	960.00	66.00	14.54	0.00	14.00
	Butyrate	960.00	66.00	14.54	0.00	13.60

*All N_2_O and N_2_ values are estimated from data in the papers and represent the cumulative production of these gases over 72 h. Values from Senbayram etal. (2012) were calculated by numerical integration from a graph of rate of N_2_O production. Soil in the studies represented were all collected from either 0–15 or 0–20 cm depth and sieved to either 2 or 5 mm. WFPS ranged from 67–70% between studies and pH ranged between 6.0 and 6.2. The only exception is Morley and Baggs (2010), who worked with slurries in an O_2_ controlled headspace. All values stated from this paper are for an O_2_ headspace concentration of 2%.*

To this end considerable effort has been expended to link activity measures such as N_2_ and N_2_O flux, ${\text{NH}}_{4}^{+}$ pools or potential denitrification rates with bacterial community structure in soil (Dandie et al., 2008; Miller et al., 2008; Kandeler et al., 2009). These efforts have proved to be largely unsuccessful with links between the ${\text{NO}}_{3}^{-}$ reducing community structure, population size, and functional measures remaining elusive. There are many potential reasons for the failure to link community and flux but two of the best candidates will act in combination and are the facultative nature of nitrate reductive processes and the complexity of the soil habitat. The facultative nature of nitrate reduction potentially means that the genes responsible for denitrification and DNRA play only a small role in determining the location of the organism that carries them. Complexity is important as in soil large variation occurs in many parameters over small distances, so links between nitrate reducing communities and environmental variables may only be apparent at much smaller scales than commonly used in soil ecological or physiological studies. In controlled experimental systems where conditions are less variable, disconnect between community, N_2_O and N_2_ production still occurs. This is despite soil in microcosm studies having been sieved and homogenized. For example, Henderson et al. (2010) found N_2_O from denitrification to significantly increase over time with C and N addition. In the same microcosms, the gene copy numbers of *nosZ* and *nosZ* mRNA transcripts were shown to increase significantly over time but there was no significant link between gene abundance or mRNA and gas flux. As such one could conclude that *nosZ *is a poor predictor of N_2_O emission as its role is to consume N_2_O.

It is important to note that there remain many methodological problems with analyzing nitrate reducing communities in soil. The diversity and abundance of the nitrate reducing community is often underestimated as a result of poor primer coverage (Green et al., 2010). Many organisms that contain nitrate and nitrite reduction genes are not necessarily denitrifiers. PCR-based analysis of these genes cannot discriminate between microorganisms that simply contain nitrate and nitrite reductases and true denitrifiers. Links between N_2_O flux, community, and environmental variables are further complicated by the ease with which denitrification genes can be transferred horizontally. In addition, establishing links between community structure and gene abundances to denitrifier rates and N_2_O emissions is problematic due to spatial variability (biotic and abiotic), and the scale these operate at in soils. Although spatial variables could be applied to all soil processes such as respiration, methane oxidation, and methanogenesis we want to address this aspect in relation to nitrate reduction, and specifically denitrification.

## SPATIAL VARIABILITY AND IMPLICATIONS FOR DENITRIFICATION

Within-soil variation in abiotic conditions will play an important role in determining the spatial arrangement of denitrification. This is especially true in the rhizosphere, where root growth and inputs can cause large changes in soil conditions over a small distance. The importance of spatial variation in abiotic factors to denitrification has been shown at field scales (Harms et al., 2009; van den Heuvel et al., 2009), but has the potential to occur on much finer scales. Disproportionately active areas or points of time are referred to as hot moments or hot spots, however, the small scale variation within soil has made identification of these difficult (Groffman et al., 2009).

On a fine scale, soil is a highly complex spatial environment which exhibits dramatic gradients in critical resources such as oxygen, carbon, nitrogen, and water, all of which interact to produce a diverse set of habitats that have proven difficult to study (Garrigues et al., 2006; Poll et al., 2006; Herron et al., 2010; zu Schweinsberg-Mickan et al., 2010). The large range of habitats is also temporally variable with alterations in the supply and utilization of chemical substrates driving further complexity in the system. This is thought to lead to the high diversity found in soil. Curtis et al. (2002) estimated that the microbial diversity of soil systems was 6400–38000 species/g compared to only 160/ml in oceans. These factors result in a system that exhibits spatial scaling over extremely small distances (Nielsen et al., 2010; Slater et al., 2010; Ruamps et al., 2011).

**FIGURE 2:**
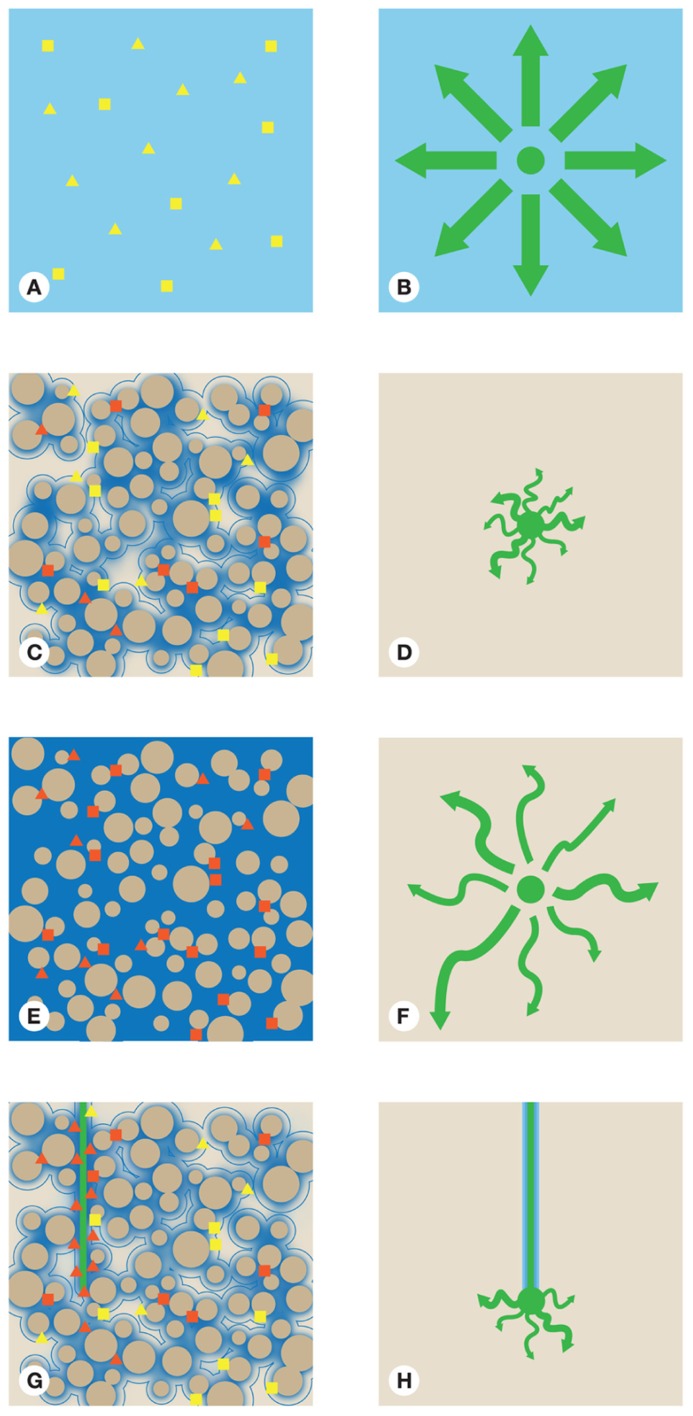
**The hypothetical location and activity of one bulk soil adapted bacterium (square) and one rhizosphere adapted bacterium (triangle)**. Bulk soil adapted organisms are assumed to be adapted for nutrient poor environments while rhizosphere organisms are assumed to be adapted for high nutrient environments in **(A)** a homogenous situation **(C)** unsaturated soil, **(E)** saturated soil, and **(G)** where a root is present. Water is represented by blue shading, the darker the the shade of blue the lower the O_2_ concentration. The distribution and movement of a resource at one point in time is shown in **(B)** a homogenous situation **(D)** unsaturated soil, **(F)** in saturated soil, and **(H)** where a root is present. The •represents the resource at its source. The larger the dot the more resource left at that point. Arrows represent the potential movement of the resource away from the source by diffusion. All diagrams with the exception of **(H)** assume a system with no flow. The length of an arrow represents how far the resource has moved. The width represents the amount of resource moving in that direction. The straighter the arrow the straighter the path the resource is taking away from its source. The two bacterial species depicted as shapes are colored with the color denoting terminal electron acceptors as O_2_ (yellow) and N compounds (red).

Unless consideration is made for spatial variability when a sampling strategy is designed there is an implicit assumption that any system is homogeneous in regards to microorganism and resource distribution, or that any variation is averaged due to the relatively large sample size taken. These may not be useful assumptions as soluble compounds move through soil by mass flow, hydrodynamic dispersion, and diffusion. In situations where water is flowing, mass flow is the main transport mechanism. A hypothetical uniform system is represented in **Figures 2A**,**B**. In such a system the spread of a soluble resource, such as organic C, from a point source (represented by the dot), in a system with no flow, would be equal in all directions with the concentration of the resource inversely proportional to the distance from the source. Concentration gradients in the resource would cause it to spread by diffusion until the resource was evenly distributed throughout the system. In such equilibrium situations denitrifying microorganisms could be expected to be randomly distributed with no regard to species and with activity dependent on a local but evenly distributed resource. For example, in **Figure 2A**, O_2_ is evenly distributed and the entire nitrate reducing community would be either active or inactive dependent on O_2_ levels, provided all other requirements are met. Also the selective drivers for community structure would be relatively constant over the area affected by the resource, giving rise to a homogeneous community structure over large distances. In reality, this situation is unlikely, although aquatic systems can approach such situations at large scales, such as in O_2_ minimum zones. Indeed, studies on such systems frequently demonstrate clear links between community structure, activity, and flux across relatively large spatial scales such as was found by Ward et al. (2009). In soil, such homogeneous situations would be rare but, by comparing two distinct spatial areas without considering the inherent variability, this assumption is commonly made.

In soil, the situation is much more complex owing to the intricate spatial structure imposed by soil particles. The drivers of soil abiotic heterogeneity are diverse with water content and soil structure often key as they influence the transport and location of many chemical compounds (Raynaud, 2010). Differences in soil particle shape and size means that no two flow paths are identical. In unsaturated bulk soil (**Figure 2C**), water is held as a film around soil particles by capillary action leaving air spaces in many pores. Which pores retain water will depend upon their size, geometry, and connectivity. Water is less affected by capillary action in the center of larger pores compared to smaller pores because of the greater distance to a soil particle. As **Figure 2D** shows the area that is affected by a resource is much reduced compared to the homogenous example in **Figure 2B**. This is in part a result of the limited medium through which diffusion and mass flow can act and the complex spatial structure created by unsaturated conditions (**Figure 2C**). This leads to highly tortuous flow paths for compounds, with the result that they move large distances without traveling far from the point source. The velocity of solutes is also affected as velocity is dependent on a diverse range of interrelated factors, including water velocity, solute residence time, soil moisture content, and hydraulic conductivity (Hillel, 1998). The situation is then complicated by the non-even packing of soil particles resulting in uneven connectivity of pore systems. This results in preferential flow of water (with associated solutes) through more connected pores and those pores with more water. These factors combine and result in large local variation in resource availability over small distances. Due to the dynamic nature of soil the nitrate reduction rates at any point in space and time will be locally dependent on the relevant factor that is limiting, either directly or through long-term control of nitrate reducer abundance in that microhabitat.

The effect of unsaturated conditions on O_2_ is depicted in **Figure 2C**. The most anoxic conditions are found in saturated pores whilst pores with air space tend to remain oxic. This has implications for nitrate reducing processes, which will be more active spatially where O_2_ is limiting. As the responsible processes are facultative, the distribution of species capable of nitrate reduction are essentially random, with organisms adapted to low-input systems (squares) more numerous as they will have a selective advantage in bulk soil. The N reducing activity of the organisms present will be driven by local environmental conditions, relying for example, on low oxygen tensions and a suitable C and N supply. This leads to a situation where only a proportion of the community capable of nitrate reduction is active at any one time. This disconnect may help to explain the inability to link community structure with flux at the scale often attempted in microcosm experiments (1–100 g) since flux measures would average over the whole sample whilst community would assess both active and inactive components of the population (Dandie et al., 2008, 2011; Wertz et al., 2009).

In saturated soil the limits on the distribution of resources is greatly reduced as mass flow and diffusion are not limited by soil water content, which would be uniformly distributed throughout the soil profile. If a resource is again added at one point in a system with no flow (**Figure 2F**), diffusion would transport the resource away from its point source and its distribution would be affected by hydrodynamic dispersion. Movement of the resource would be enhanced through wider pores. The movement of the resource would also be affected by tortuosity moving further from the source in areas where the flow path is straighter. The distribution of resources would be much less restricted than in unsaturated soil and as a result more uniform in an equivalent volume. Microorganisms are again randomly distributed in regards to species and just as in unsaturated systems, microorganisms that are adapted to resource poor conditions are more numerous (**Figure 2E**). The O_2_ content of saturated soil will be much more limited than in the unsaturated soil. This could be expected to result in a much more even distribution of nitrate reduction, as long as no other resources are limiting. In soil this is an unrealistic assumption as different compounds exhibit varying abilities to move through soil. This is a consequence of the different abilities of compounds to adsorb on to soil particles and differences in how they are used by soil microorganisms. This varying ability to move is often described by a compounds diffusion coefficient. With the exception of wetland areas, terrestrial soil systems are rarely saturated for long periods and soil water content can change with weather conditions, irrigation regimes, and variation in drainage. Saturated soil situations are therefore unlikely to be stable enough to be able to demonstrate links between community, activity, and environmental conditions. In stable saturated environments such as estuarine sediments, links have been found between the community, activity, and environmental factors (Ward et al., 2009; Abell et al., 2010), potentially as result of reduced spatial heterogeneity.

Roots introduce an extra level of spatial complexity. Within the rhizosphere, resources originate from the plant roots but the flow of water is also driven toward the root by plant uptake (**Figure 2H**). Diffusion of resources from plant roots act against the flow of water, hence limiting the distance over which they travel and restricting the spatial extent of the rhizosphere. Specialist microorganisms capable of maximizing the use of the high concentration of resources therefore dominate within the rhizosphere (triangles) and are particularly abundant around the root tip, where the greatest input of resources occurs. In contrast, microorganisms adapted to low-input systems are found preferentially in the bulk soil (**Figure 2G**), though may be present in low-input areas of the rhizosphere. Nitrate reduction is likely to be high in the rhizosphere, where C resources are higher and the O_2_ content potentially lower because of root respiration. This can be seen from studies that have compared systems with and without plants (Hojberg et al., 1996; Mahmood et al., 1997), where rates of denitrification have been found to be higher in planted systems. Rhizodeposition is known to select for distinct and more active microbial communities than that of the bulk soil (Marschner et al., 1982, 2004; Paterson et al., 2007; Hartmann et al., 2009). Root inputs are transient in any given location and the microbial response to variables such as root-derived C flow in the rhizosphere is very rapid (minutes to hours; Boddy et al., 2007; Hill et al., 2008), potentially creating an unstable habitat as the root passes through an area of the soil. It has been suggested that this is associated with the selection of fast growing organisms (Blagodatskaya et al., 2004). This larger and more active microbial community has the potential to affect nitrate reduction and may be one of the reasons why studies have found exudates to stimulate denitrification (Hojberg et al., 1996; Mahmood et al., 1997; Mounier et al., 2004; Henry et al., 2008). As plants can alter a number of conditions controlling denitrification it is not possible to easily determine which plant altered conditions have the greatest affect on nitrate reduction. Studies focusing on individual factors that can alter nitrate reduction in the rhizosphere are needed in order to fully understand the effects of roots on nitrate reducing communities and rates of this process.

Due to essential limitations of sampling size, both functional gene and gene expression studies focus on averaging the contribution of community members across a large range of habitats. To truly link community structure and function, and understand the variability of responses to soil conditions requires sampling to occur at a relevant spatial scale. This may require analysis at a maximum of aggregate scale perhaps using a framework supplied by non-invasive methods, such as X-ray CT scanning combined with modeling to estimate soil conditions and nutrient flow (Tracy et al., 2010; Zygalakis et al., 2011). The temporal and spatial complexity inherent within soil means that at any time there will be a range of spatially segregated habitats optimal for nitrate reduction through either classical denitrification or DNRA. The proportion of habitats optimal for either process or for that matter other anaerobic or aerobic process, such as nitrification, will be highly variable. This means that connections between community structure and activity are unlikely ever to be clear, without studies focusing at a spatial and temporal scale relevant to the organisms concerned.

### SCALE

As described above, spatial and temporal scales are two potentially critical factors in any relationship between N_2_O, N_2_, and the microbial community. Spatial patterns in ecology have been recognized as being affected by the scale over which an organism is studied, and is intrinsically linked to the patchiness of “habitats” (Levin, 1992). The link between the products of denitrification or DNRA and habitat is likely to be determined by the scale at which the question is posed. For example, linking a bacterial species or community structure to factors at a field scale may identify a link to wet areas or, if assessed within only wet areas, high carbon concentration or if assessed at an aggregate scale the surface of soil particles of a particular size or composition. Thus, to fully understand the dynamics of a nitrate reducing community there is a requirement for investigation not only at field scales but also at the scale over which a bacterial colony or community operates. In the case of microorganisms this may require assessment at a millimeter or sub millimeter scale, as the high spatial variability of soil conditions creates a very patchy set of habitats. For example, both Chenu et al. (2001) and Nunan et al. (2003) demonstrated that bacterial density varied at scales as small as 1 mm. Temporal scales are also critical as bacterial communities have been shown to shift rapidly with changing resources, such as the addition of labile C compounds (Cleveland et al., 2007; Jones and Murphy, 2007).

Scale has strong implications for understanding the link between community structure and function, as the variables which affect nitrate reduction processes and the communities responsible are scale dependent. If community measures and associated fluxes of N_2_O or N_2_ are quantified at different scales, they are unlikely to connect to each other. This will either prevent the demonstration of links between community, flux, and abiotic variables or provide a link derived by random chance. Any measure of the flux and community across an area must sample as many of the potentially different habitats as practically possible with a suitable level of replication, to allow trends to be seen despite soil variability. Even in a small scale sample of a few grams this will mean that the assessment of community structure and activity will be occurring across several micro-habitats. In order to truly understand the factors affecting the ecology of nitrate reducing communities and hence their drivers, these microorganisms need to be investigated at extremely small scales or up scaled using representative artificial systems.

Knowledge of the spatial structure of soil communities over relatively large distances is still critical, as it represents a more manageable scale for developing strategies to mitigate N_2_O emissions. Community structure provides an insight into the effects of land use on microbial processes, but a link between the structure of the community and flux of N_2_O or N_2_ at any one sampling point would likely fail. Over large distances soil communities can often display spatial structure, with a number of examples demonstrating links between community and its position. Examples include effects on N cycling organisms, which operate over many scales but are commonly linked to shifts in land use, soil conditions such as pH or life history strategies (Franklin and Mills, 2009; Bissett et al., 2010; Enwall et al., 2010; Berner et al., 2011; Bru et al., 2011; Dequiedt et al., 2011; Griffiths et al., 2011; Keil et al., 2011).

In a rhizosphere context, comparisons between bulk and rhizosphere soils are generally performed at a coarse scale when compared to the size of microorganisms responsible for nitrate reduction. There appear to be no direct measurements of variation in either denitrification or DNRA across the rhizosphere, yet there is the potential for variation in these soil functions, which can be inferred from knowledge on the spatial variation of factors that affect these processes. As a result of the small scale of changes in the rhizosphere, studies addressing variation in resources observed this have been forced to average conditions across distance bands (zu Schweinsberg-Mickan et al., 2010). This approach averages the environmental conditions found in many micro-habitats with a high potential to group areas of high activity with areas capable of much lower activity, leading to misleading links between N_2_O fluxes and biotic and abiotic conditions within soil.

## FUNCTIONAL DIVERSITY AND NITRATE REDUCTION

The effects of spatial variability and environmental variables on nitrate reduction cannot be fully understood without a better understanding of the dynamics of the microbial communities responsible. The wide range of organisms able to carry out denitrification means that they exhibit a high level of both functional diversity and functional redundancy across a range of phylogenetically diverse organisms, with a wide range of environmental tolerances (Zumft, 1997). Less is known about the organisms involved in DNRA, but they are likewise believed to be relatively ubiquitous (Smith et al., 2007). The high degree of functional diversity and functional redundancy has led to the suggestion that short-term factors, that activate functional genes, or limit reactions (such as insufficient substrate) may be more important determinants of denitrification rates than the underlying microbial community (Wallenstein et al., 2006).

Nevertheless, community dynamics will play a role especially under conditions where the underlying community structure is fluid. Additionally, it is important to note that it is likely that the dominant ${\text{NO}}_{3}^{-}$ reducers will vary under differing environmental conditions. However, the full extent of the community structure and dynamics on governing ${\text{NO}}_{3}^{-}$ reduction rates is not well understood. The community can affect the ability to use and respond to different resources. Paterson et al. (2011) showed that where plants were absent for a period of time the diversity of organisms that were able to utilize plant inputs decreased, and Eilers et al. (2010) found that communities from different soils responded differently to the inputs of glucose and other C compounds. The community present may play an important role in determining which resources are able to be utilized rapidly especially if the input of a resource is transient, such as rhizodeposition. It is unclear how different species of microorganisms utilize different resources and how this affects the gene regulation of each species. As community composition is likely to be important, a greater knowledge of nitrate reducing taxa and their life histories would help to elucidate the effects of community. In reality, this is impractical because of the extremely large number of individual microbial species found in soil.

Nitrate reducers are generally treated as a single homogeneous group, even though the niche of individual species (range of conditions tolerated) is unlikely to be defined entirely by the genes for the facultative processes of denitrification or DNRA. A niche will instead be a cumulative effect of many genes and how they are regulated between species. Indirect evidence for this can be seen in the varying conditions required to culture different denitrifiers (Nokhal and Schlegel, 1983; Lang et al., 2007), though it remains unclear if these requirements translate into the soil environment. The denitrification genes are likely to only define the ability to persist and to be competitive under anaerobic conditions. The functional diversity of denitrifiers has the potential to limit the effects of spatial variability of soil conditions on nitrate reduction. This functional diversity will make nitrate reduction resilient to changing conditions, and will complicate efforts to link gene expression to environmental conditions.

A possible exception to the lack of selective effects of denitrifier functional genes has been observed with *nirK* and *nirS*. These two genes code for enzymes with the same function, nitrite reduction, but differ in the structure of the nitrite reductase enzyme. The reductase encoded by *nirK* contains copper while the reductase coded by *nirS* contains a heme center. These two genes have been found by several studies (Enwall et al., 2010; Keil et al., 2011) at field scales, to be dominant in spatially distinct areas. This lack of overlap between denitrifiers containing *nirK* and *nirS* has been attributed to the need for copper to support *nirK* “activation” in contrast to *nirS*. The separation of location between bacteria containing *nirK* and *nirS *may provide evidence that facultative functional genes can play a role in determining the location of organisms despite the high functional diversity found in denitrifiers. Studies on *narG*, *napA*, and *nosZ*, which is another copper based enzyme, have not found similar separation between these genes (Enwall et al., 2010; Keil et al., 2011). This is likely due to these genes encoding different enzymes in the denitrification pathway and for the potential for organisms to contain more than one of these functional genes.

### FUNCTIONAL REDUNDANCY

Stability in nitrate reducing processes may also be linked to functional redundancy. If there is sufficient redundancy in a system the removal of a number of species will have little effect on function, as their loss will be compensated for by species with similar capabilities. Thus, function will be largely resistant to environmental change. The biodiversity of an ecosystem is believed to play a role in the stability of an ecosystem’s function (Torsvik and Ovreas, 2002; Balvanera et al., 2006), with stability increasing with the species richness of organisms capable of carrying out the function in question. There remains much uncertainty about the relationship between diversity and function with studies finding conflicting results. For example, Girvan et al. (2005) found that C mineralization was more resistant to benzene perturbation in more diverse soils, whilst Griffiths et al. (2004) and Wertz et al. (2006) found no link between diversity and function. The study by Wertz et al. (2006) assessed the effects of biodiversity by comparing N_2_O emissions from soils with decreasing species richness and found N_2_O emissions to be relatively stable, which was attributed to functional redundancy. These studies represent the potential for variation at a relatively large scale, having used samples of homogenized soil. Functional redundancy may also operate at smaller scales with variation in soil environmental conditions. Zhang et al. (2010) provided evidence for this and found the stability in respiration of a complex substrate was linked to micro-scale habitats, although the effect varied with plant cover (land use history) and the stress imposed (copper and heat).

### MECHANISMS OF COEXISTENCE OF SPECIES

Mechanisms that allow for the maintenance of species richness, and hence high functional diversity and redundancy, are important in allowing microbial activity and associated nitrate reduction to occur across the spatial heterogeneity found in soil. Increased knowledge on what segregates microorganisms and how they coexist, will help with understanding how functional diversity is maintained in soil, how communities adapt to different conditions and the associated consequences for nitrate reduction activity.

One important mechanism of coexistence is the differing tolerances and resources required by different species. This can be described by the ecological niche concept, which describes the range of conditions under which a particular species may exist. A difference in niches between species is one of a number of factors that have been used to explain the co-existence of organisms in ecology (MacArthur and Levins, 1967). Distinct niches reduce interspecific competition and thus competitive exclusion. Work performed in culture by Salles et al. (2009) found that different bacterial denitrifiers consumed different low molecular weight carbon compounds, which resulted in different rates of denitrification. They suggested that this could represent different ecological niches in their system. In soil systems, carbon will act as only one of the diverse factors that constitute a species niche. Work done by Paterson et al. (2007), in an artificial system, also found that members of the microbial community utilize different C compounds to different extents. Further to this, Paterson et al. (2008) found that the chemical forms of plant input were important in maintaining microbial community structure in soil, as the fate of C was dependent upon the form and the transfer between microbial groups was slow.

The spatial heterogeneity in soil and wide range of resources provided, allows for the co-existence of many species with a wide range of niches. This is aided by both the variety of basic low molecular weight C compounds affecting species differently, and soil transport mechanisms creating a diverse patchwork of these C compounds over small distances in soil. The resulting high species richness helps to maintain the high functional diversity of denitrifiers and the presence of microorganisms capable of DNRA, in a situation relatively free of competition. Whether these two functions compete with each other for resources such as ${\text{NO}}_{3}^{-}$ and C under limiting conditions will depend on the extent to which the species niches of organisms capable of these processes overlap. In reality, the niche concept has limited success in explaining coexistence of species. In some systems it is believed to explain the overall patterns in diversity well, while in others it is unable to do so (Brokaw and Busing, 2000; Elliott and Mariscal, 2001). Species niches often overlap, so in systems where organisms utilize the same resources other factors will play a role in determining which species coexist, the most important of these are interspecific competition and immigration. The niche concept may be useful in understanding the location of organisms and could be useful in understanding the functioning of microorganisms, though much more work needs to be done before this concept can be applied reliably in soil. The difficulty of applying classical ecological theory to soil is compounded by the lack of a definite species definition and processes such as horizontal gene transfer, which can cause rapid changes in the accessory genome of microorganisms (Prosser et al., 2007).

### GENE ACTIVITY MEASURES

The assessment of which organisms are expressing nitrate reducing functional genes may prove to be more helpful in understanding the link between communities and nitrate reduction under any given set of conditions. Focusing on gene expression provides a means of identifying the organisms actively involved in denitrification and DNRA assuming post-transcriptional control is insignificant. Simply assessing the functional gene complement of the community will inevitably create bias as it will measure organisms that are both active and inactive. In pure culture, mRNA has been used successfully in conjunction with NO, N_2_O, and N_2_ measurements to investigate the response of single factors on denitrification (Bergaust et al., 2010). Pure culture experiments represent a much simpler system than soil, akin to the homogenous example shown in **Figures 2A**,**B** and many of the studies on the gene regulation of denitrification has focused on the responses of the model organism *P. denitrificans*. There is however evidence that gene regulation varies between organisms (Bergaust et al., 2011), making a link between gene regulation to communities more complicated in soil. Only recently has analysis of mRNA become common in soil systems due to issues surrounding its stability, with a half-life of circa 1.3 min at 37°C (Saleh-Lakha et al., 2011), and may represent the best chance of linking structure and function in environmental systems. Recent studies in soil which have attempted to link the expression of *nirK *and *nirS *with the production of N_2_O, have found no significant relationship between mRNA abundance and N_2_O production (Henderson et al., 2010; Dandie et al., 2011). There are still relatively few studies linking gene expression to N_2_O emissions and environmental variables. Perhaps *NorB* expression or the *NorB/NosZ* ratio might prove to be better functional indicators of net soil N_2_O emissions. However, these links are likely to be problematic to discover as there are a number of methodological limitations, as described below.

## METHODOLOGICAL LIMITATIONS

Studying processes at the sub millimeter scale at which soil variability can exist presents methodological problems. Depending on the question posed a number of measures are needed to understand the links between community, gas flux, and environmental variables. These can include C and N concentrations and form, water content, pH, N_2_O, and N_2_ concentrations and community measures. Analysis of all these variables is difficult at fine aggregate scale resolutions, though advances in micro-sensors may offer part of the solution. Micro-sensors exist for a range of compounds including, NO_x_, ${\text{NH}}_{4}^{+}$, pH, O_2_, and N_2_O (Revsbech, 1989; Andersen et al., 2001). Micro-sensors have been successfully used to determine N_2_O profiles from soil (Elberling et al., 2010) and in fresh water sediment where Stief et al. (2010) used them to determine N_2_O concentration gradients across 25 mm of sediment. As with any methodology there are limitations to the use of micro-sensors. While measures of N_2_O are important, it is also useful to identify the sources of N_2_O as it can be produced by a number of other processes including NH_3_ oxidation as well as DNRA and denitrification. ^15^N isotope labeling techniques provides a means for identifying the active soil process and has been used at small scales in freshwater sediment by Stief et al. (2010). They were able to quantify ${\text{NH}}_{4}^{+}$ production by DNRA at a resolution of 1–2.5 mm. This was achieved by adding ^15^N labeled ${\text{NO}}_{3}^{-}$ to sediment and quantifying ^15^ ${\text{NH}}_{4}^{+}$ that had been trapped on a polyacrylamide gel inserted into the sediment. Care must be taken when sampling small areas as the act of placing sensors or gels into the soil will alter the soil structure and hence the small scale variability in conditions that are needed to link community and flux. Factors such as bulk density, nutrient flow, gas diffusion, and community composition may all be altered by the disruption of soil structure. As with any small scale measure of soil variability there will be problems with identifying the exact point in soil that is being measured, as it will need to be identified through an opaque medium. Methods of imaging soil such as X-ray CT scanning, described above, may provide a solution for this but this can only derive the physical structure of the matrix and cannot resolve the processes or associated microbial communities.

Measures of N_2_O at these small scales may prove to be of limited use if they cannot be linked to the associated environmental and community variables that drive the production of this gas. As well as micro-sensors there are a number of sensitive analytical techniques such as high pressure liquid chromatography (HPLC), gas chromatography (GC), and bioassays, that allow the measurement of C and N at low concentrations and in some cases allow the identification of the form of C and N. These methods have been used to quantify compounds in soil and rhizodeposits from plants (Jones and Darrah, 1995; Fischer et al., 2010). The greatest constraint to using these methods is the ability to retrieve and analyze samples from areas of soil that are potentially below 1 mm in diameter, and ensuring that any sample is accurately linked to the area of measured N_2_O production.

Community measures must also be linked to flux and environmental variables, and suffer from the same issues of sampling at small scales. A study by Ranjard et al. (2000) was able to use aggregate washing to separate bacteria from inside and outside aggregates and bacteria from different sizes of aggregates, which could then be used in community analysis. Other studies have also used fractionation to distinguish between communities on different sized soil particles such as Kandeler et al. (2000). These methods group all particles of a particular size category from a sample together and may not offer a suitable level of resolution to capture the small scale spatial variability in soil created by differences in nutrient transport. There is however evidence that small subunit 16S rDNA can be amplified from smaller samples, a study by Kotani-Tanoi et al. (2007) was able to amplify bacterial DNA from individual soil particles.

PCR-based techniques provide a powerful tool for the analysis of microbial communities. The population of nitrate reducers can be estimated using RT-PCR, which gives a highly sensitive way of measuring functional gene copy number. Diversity measures can be estimated from techniques such as T-RFLP and DGGE and targets can be amplified from relatively small amounts of template DNA. There are a number of considerations when using PCR-based techniques, which have been reviewed by Hirsch et al. (2010). Problems include poor primer coverage, which is a common issue in molecular ecology. Regular updating of primers is essential together with an understanding that a significant proportion of any target group may be excluded from analysis. Denitrification genes are particularly problematic, with many primers unable to amplify targets from significant proportions of the denitrifier community, because of mismatches with commonly used primers (Green et al., 2010). Any measures of the diversity of nitrate reducers or quantification of the nitrate reducing genes are therefore likely to be underestimates. Primers targeting *nirS* have been found to be particularly problematic (Throback et al., 2004). The under representation of community components through primer selection may mean that links to important abiotic variables are missed. PCR bias also plays a role in affecting which taxa are amplified (Polz and Cavanaugh, 1998). While variation in extraction efficiency and presence of inhibitors can affect conclusions made using RT-PCR unless a relative approach is taken to account for variation between samples (Daniell et al., 2012).

### FUTURE PERSPECTIVES

If environmental variables and soil communities are to be linked to the products of denitrification and DNRA, work is needed on both converting results from culture to soil systems, and on investigating soil systems at smaller scales. Results in simple systems, such as pure culture, are able to link functional genes, environmental variables, and the products of denitrification allowing useful insights into the factors that control denitrification and the underlying biochemistry. There are however a limited number of nitrate reducers that are culturable and there exists the potential for variation in responses between taxa. Results from culture are not always directly comparable to more species diverse and complex soil environments. There is a need to understand if the nitrate reducers investigated in culture are representative of the bulk of the denitrifier community found in soil, and how increasing soil diversity affects their responses. More detailed studies of simple systems are still important. These types of systems are likely to provide information that will improve the understanding of the role of the microbial community on nitrate reduction and how it can affect the response of a soil community to environmental factors.

To provide this information there is a need to investigate nitrate reduction at smaller scales both with simple and complex communities. This will allow insight into whether sampling regime and scale can play a role in determining links between environmental variables, the products of nitrate reduction, and the microbial community. Measures of environmental variables at pore scales are at a size more appropriate to the communities that drive nitrate reduction and may provide insight into the links between community, abiotic factors, and the flux through denitrification and DNRA, that are elusive at larger scales.

## CONCLUSION

The environmental controls on denitrification are relatively well established. Both soil and culture studies provide evidence of C, N, O_2_, and pH driving both the rates and products of denitrification. The controls on DNRA while similar are not as well understood. In soil a wide variation in N_2_O production occurs between studies which may in part be accounted for by the varying experimental conditions, microbial communities, and soils used. Despite the possible importance of the nitrate reducing community to the rates and products of nitrate reduction, no clear links have been found between the community composition, the flux of gases produced by either denitrification or DNRA, and environmental conditions. These links are evident in culture and environmental studies covering relatively homogenous habitats. The relationships break down in studies of highly complex systems such as soil, regardless of whether assessment is made using functional gene or mRNA measures. Difficulty in proving these connections is likely to be driven by the variability in micro-habitats over small distances, which can alter the availability of resources and the composition of the communities that control nitrate reduction. This resource variability potentially drives the coexistence of microorganisms and supports high levels of functional diversity in the nitrate reducing community, which will impact their response to the small scale spatial variability of resources in soil. Additionally, it is not known if nitrate reduction genes have an important role in driving general soil community structure, although this is likely to be restricted with facultative processes such as this. Methodological limitations, primarily in N_2_O and N_2_ measurements and community analysis, currently limit studies at the scale required to demonstrate clear links between community and environmental variables. If nitrate reduction is to be understood a greater understanding of the communities capable of nitrate reduction is needed, and how they link to the current knowledge on gene regulation.

## Conflict of Interest Statement

The authors declare that the research was conducted in the absence of any commercial or financial relationships that could be construed as a potential conflict of interest.

## Acknowledgments

This work was supported with a NERC CASE studentship grant. The James Hutton Institute is financially supported by the Scottish Government Rural and Environment Science and Analytical Services Division. We thank both Lionel Dupuy and Roy Neilson, internally to the James Hutton Institute, external reviewers for helpful comments during review, and Cavan Convery for help with the diagrams.
